# Study on the spatial distribution patterns of histone modifications in Hippo pathway genes

**DOI:** 10.52601/bpr.2021.200042

**Published:** 2021-02-28

**Authors:** Wenxia Su, Dimeng Zhang, Qiang Zhang, Qianzhong Li

**Affiliations:** 1 College of Science, Inner Mongolia Agriculture University, Hohhot 010018, China; 2 Laboratory of Theoretical Biophysics, School of Physical Science and Technology, Inner Mongolia University, Hohhot 010021, China; 3 The State Key Laboratory of Reproductive Regulation and Breeding of Grassland Livestock, Inner Mongolia University, Hohhot 010070, China

**Keywords:** Embryonic stem cell, Hippo pathway, Histone modification, Spatial distribution pattern

## Abstract

Hippo pathway can regulate cell division, differentiation and apoptosis, and control the shape and size of organs. To study the distribution patterns of histone modifications of Hippo pathway genes in embryonic stem cells is helpful to understand the molecular regulation mechanism of histone modification and Hippo pathway on stem cell self-renewal. In this study, 19 genes of Hippo pathway including YAP, TAZ, LATS1/2, MST1 and SAV1, and eight histone modifications in embryonic stem cells were chosen to study the spatial distribution patterns of histone modifications. It was found that there were obvious type specificity and the location preference of target regions in the distributions of histone modifications, and H3K4me3 and H3K36me3 played the most important regulatory roles. Through the correlation analysis of histone modifications, a histone modification functional cluster composed of H3K4ac, H3K4me3, H3K9ac and H3K27ac was detected in YAP. In addition, the spatial distribution patterns of histone modifications in Hippo pathway genes were obtained, which provided a new theoretical reference for elucidating the mechanism of histone modifications regulating the gene expression of Hippo pathway, and for revealing the molecular regulatory mechanism of histone modifications affecting the self-renewal of embryonic stem cells by regulating the Hippo pathway.

## INTRODUCTION

Hippo pathway is regulated by cell density, cell contact, and related to cell proliferation (Huang *et al*. 2005; Kim *et al*. 2011; Mori *et al*. 2014), apoptosis (Lee and Yonehara 2012) and organ size regulation (Camargo *et al*. 2007; Zhao *et al*. 2007). Hippo pathway is commonly used to coordinate organ development and control tissue homeostasis by regulating cell proliferation, apoptosis, migration and differentiation. Hippo pathway also regulates stem cell self-renewal and tissue regeneration (Mo *et al*. 2014; Ramos and Camargo 2012). Hippo pathway is composed of a series of kinases including transcription coactivators and DNA binding proteins. Although Hippo pathway was first found in *Drosophila*, it is also highly conserved in mammals. In mammals, Hippo pathway is mainly composed of upstream sterile 20 like kinase (MST1/2) and its scaffold protein Salvador (SAV), midstream tumor suppressor (LATS1/2) and its regulatory protein MOB, and downstream transcription effector Yes-associated protein (YAP) and transcriptional coactivator with PDZ binding motif (TAZ) (Chen *et al*. 2019). At high density, the Hippo signaling pathway is opened, and the protein kinase MST1/2 activates LAST1/2 through phosphorylation. YAP is highly phosphorylated and bound with 14-3-3 protein and remains in the cytoplasm, thus inhibiting the transcription and expression of YAP downstream genes and inhibiting cell proliferation. On the contrary, under low density conditions, Hippo pathway is inhibited. YAP/TAZ complete the dephosphorylation process, and cannot bind with 14-3-3 protein, and then enter the nucleus to bind with TEAD, inducing downstream gene transcription expression and promoting cell proliferation (Lei *et al.* 2008). At first, the research on Hippo pathway mainly focused on the control of tissue and organ size. In recent years, studies have confirmed that Hippo pathway has the function of regulating stem cell self-renewal and tissue regeneration.

Embryonic stem cells are the source of developing embryos, fetuses and final adult tissues. Human embryonic stem cells rely on different signals for self-renewal: they depend on the signal of fibroblast growth factor and the balance between transforming growth factor beta (TGF-β) and bone morphogenetic proteins (BMPs) (Darr and Benvenisty 2006; Xiao *et al*. 2006). Transcriptional regulation has also been proved to be a key regulator of self-renewal and differentiation of embryonic stem cells, because forcing the expression of various transcription factors can reprogram differentiated tissues into pluripotent stem cells with self-renewal ability (Evans 2011). In stem cell biology, the study of Hippo pathway provides clues for elucidating the development process (Fu 2017; Pan 2010; Wang *et al*. 2017; Yu and Guan 2013). The study of YAP and TAZ reveals the role of these transcription coactivators in regulating the self-renewal and differentiation of embryonic stem cells. Knockout of TAZ and retention of Yap in embryonic stem cells will lead to the loss of self-renewal ability of embryonic stem cells and differentiation into neuroectoderm. When TGF-b receptor is inhibited, the same phenomenon will occur. On the contrary, LATS2 can inactivate TAZ, and knockout of LATS2 can promote the production of induced pluripotent stem cells (Qin *et al*. 2012).

At present, there have been many studies on Hippo pathway. It is clear that the Hippo pathway and its effectors YAP and TAZ play a key role in determining cell fate, proliferation and regeneration of embryonic stem cells. However, the upstream regulatory factors of Hippo pathway, the interaction mechanism between Hippo pathway and other embryonic stem cell regulatory pathways, and regulatory factors remain to be solved. The self-renewal process of embryonic stem cells is also regulated by many factors including transcription factors and histone modification (Bhanu *et al*. 2016; Feng *et al*. 2019; Su *et al*. 2016a, b; Su and Li 2015). In this study, the data of eight most common histone modifications were used to study the spatial distribution patterns of histone modifications on 19 genes of Hippo pathway in human embryonic stem cells. Through this study, we elucidated the regulatory mode of histone modifications on Hippo pathway genes, and then provided a new theoretical reference for revealing the regulatory mechanism of histone modifications affecting the self-renewal process of human embryonic stem cells by regulating the expression of Hippo pathway genes.

## RESULTS AND DISCUSSION

### Distributions of histone modifications in the flanking regions of transcription start sites of Hippo pathway genes

The normalized read counts of histone modifications in the flanking regions of transcription start sites (TSS ± 2000 bp) were calculated to obtain the distributions of histone modifications in the Hippo pathway genes, as shown in Table 1.

**Table 1:** The distributions of histone modifications in the Hippo pathway genes

	H3K4ac	H3K4me3	H3K9ac	H3K9me3	H3K27ac	H3K27me3	H3K36me3	H3K79me1
DCHS1	5.36	13.67	6.12	3.02	6.73	6.16	3.17	5.10
DCHS2	3.44	2.88	4.32	2.62	2.89	1.32	3.17	4.31
FAT1	3.02	2.52	2.52	2.41	3.53	3.96	18.68	6.66
FAT2	4.26	0.36	5.04	1.81	3.85	5.72	2.82	5.88
FAT3	2.47	1.08	1.44	4.03	4.17	4.40	2.11	3.92
FAT4	7.29	173.70	17.65	4.43	10.58	38.75	4.23	10.58
FRMD6	5.09	0.72	1.08	2.62	4.49	2.64	2.47	3.92
WWC1	6.60	82.71	16.57	2.62	10.58	7.49	2.11	9.80
NF2	5.91	78.76	17.29	2.21	10.26	3.08	3.17	7.05
MST1	4.12	7.19	7.20	2.82	2.57	11.01	23.97	7.45
SAV1	2.61	0.72	3.60	3.42	4.49	1.32	6.34	4.70
LATS1	3.30	2.16	3.60	2.01	3.53	0.00	4.58	2.35
LATS2	2.61	1.08	2.16	4.03	1.92	4.84	11.28	5.10
TAZ	3.02	46.39	8.29	1.01	8.66	1.76	2.11	4.70
TEAD1	5.91	112.2	36.39	2.62	14.75	9.25	2.11	4.70
TEAD2	5.22	25.89	8.65	4.02	13.47	3.96	13.75	6.27
TEAD3	4.95	7.19	6.49	4.23	13.79	4.84	22.91	5.49
TEAD4	11.55	155.00	40.71	4.03	28.22	7.04	5.29	15.68
YAP	9.76	192.76	85.02	1.61	73.12	7.49	3.52	7.45
The DNA regions (TSS ± 2000 bp) were divided into 20 bins of 200 bp. Firstly, the normalized read counts of each histone modification in each bin were calculated, then the total read counts for all bins were calculated.								

According to the data in Table 1, the distributions of different histone modifications on different genes are quite different, indicating that the distributions of histone modifications have obvious gene specificity. For example, H3K4me3 is distributed as high as 173.70 in FAT4, but only 0.72 in FRMD6. At the same time, the three histone modifications with the largest distributions of each gene were summarized and extracted, as shown in Table 2. H3K4me3 and H3K36me3 account for the largest proportion of the most distributed histone modifications in all genes. Thus, we can conclude that H3K4me3 and H3K36me3 are the two most important histone modifications that regulate the expression of Hippo pathway genes in the flanking region of gene transcription start sites.

**Table 2:** The order of three histone modifications with the largest distributions in each gene

	1	2	3
DCHS1	H3K4me3	H3K27ac	H3K27me3
DCHS2	H3K9ac	H3K79me1	H3K4ac
FAT1	H3K36me3	H3K79me1	H3K27me3
FAT2	H3K79me1	H3K27me3	H3K9ac
FAT3	H3K27me3	H3K27ac	H3K9me3
FAT4	H3K4me3	H3K27me3	H3K9ac
FRMD6	H3K4ac	H3K27ac	H3K79me1
WWC1	H3K4me3	H3K9ac	H3K27ac
NF2	H3K4me3	H3K9ac	H3K27ac
MST1	H3K36me3	H3K27me3	H3K79me1
SAV1	H3K36me3	H3K79me1	H3K27ac
LATS1	H3K36me3	H3K9ac	H3K27ac
LATS2	H3K36me3	H3K79me1	H3K27me3
TAZ	H3K4me3	H3K27ac	H3K9ac
TEAD1	H3K4me3	H3K9ac	H3K27ac
TEAD2	H3K4me3	H3K36me3	H3K27ac
TEAD3	H3K36me3	H3K27ac	H3K4me3
TEAD4	H3K4me3	H3K9ac	H3K27ac
YAP	H3K4me3	H3K9ac	H3K27ac

**Figure 1:**
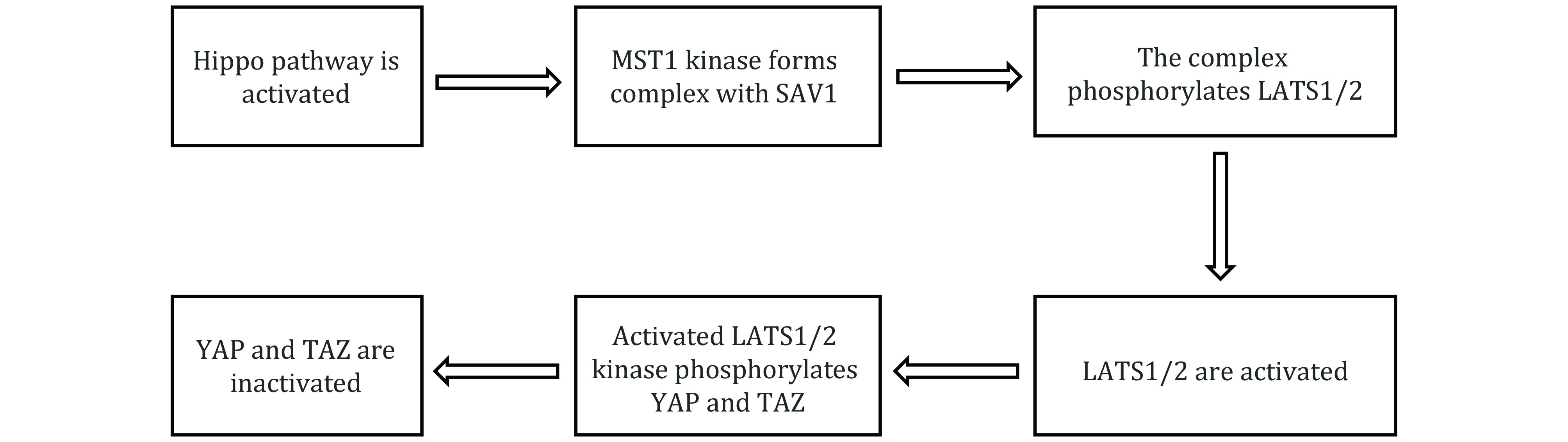
The main regulatory pathway of Hippo pathway

Previous studies have shown that the kinase cascade is the key to Hippo signal transmission. MST1 kinase forms complex with SAV1, and then phosphorylates LATS1/2. Activated LATS1/2 kinase phosphorylates YAP and its collateral homologue TAZ to inactivate them and inhibit the transcription of TAZ and YAP. On the contrary, unphosphorylated TAZ and YAP enter the nucleus to bind with TEAD1-4 or other transcription factors, and then induce the expression of the genes that promote proliferation and inhibit apoptosis (Mo *et al*. 2014), as shown in Fig. 1.

**Figure 2:**
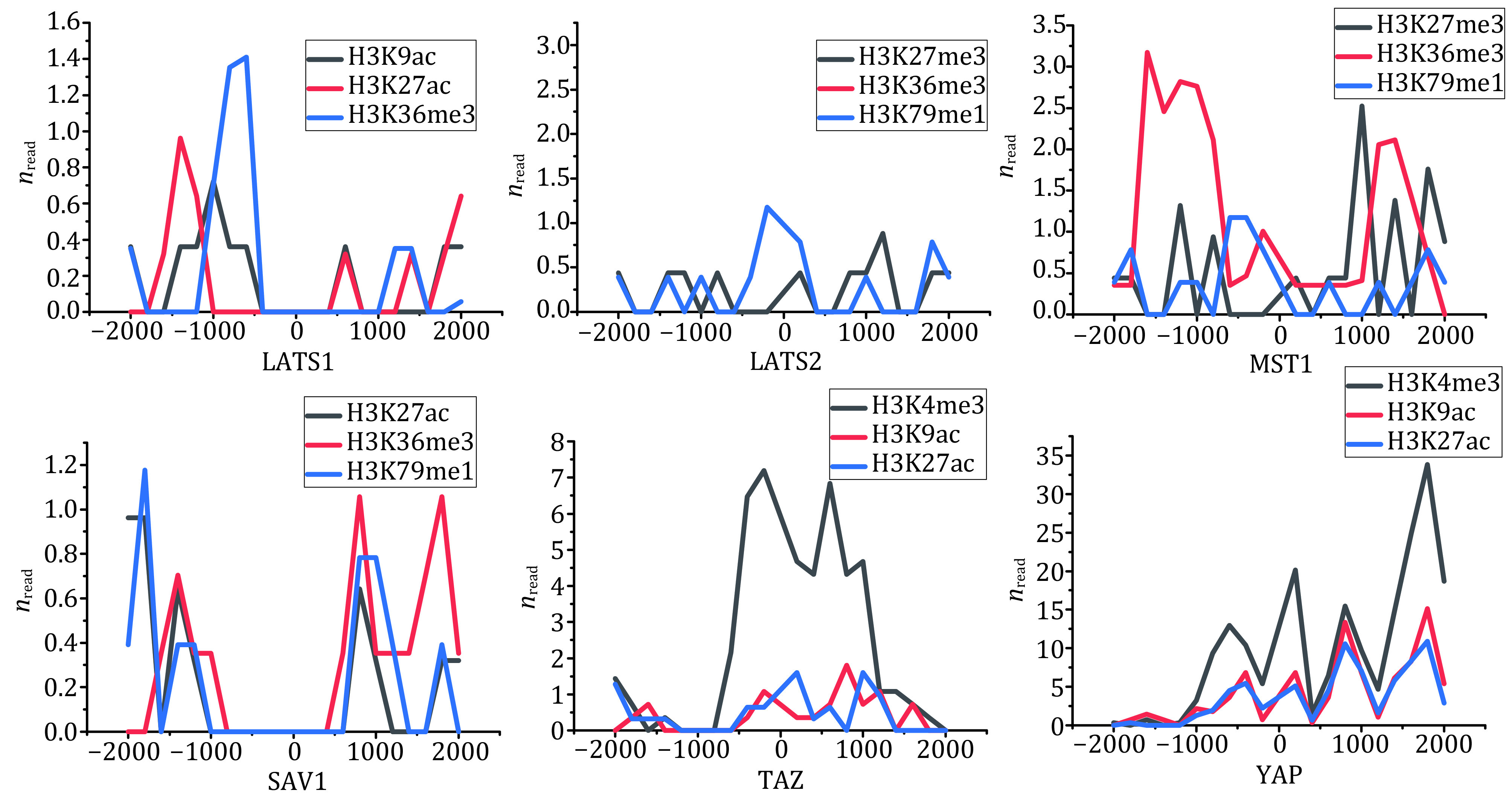
The histone modification profiles of the core genes in Hippo pathway. The three lines represent the three histone modifications with the most distributions on the core genes, respectively

Therefore, we selected the core genes YAP, TAZ, LATS1/2, MST1 and SAV1 in the Hippo pathway to obtain and analyze the distribution profiles of the three histone modifications with the most distribution on the core genes, as shown in Fig. 2. It can be concluded from Fig. 2 that, in LATS1, H3K36me3 is the most abundant histone modification, mainly located in the upstream region away from the TSSs (>500 bp); In LATS2, H3K36me3 is the most abundant histone modification, and located in the narrow region near the TSSs (<500 bp); In MST1, H3K36me3 is the most frequently distributed histone modification, and it is more distributed in the upstream and downstream regions far away from the TSSs (>1000 bp); In SAV1, H3K36me3 is the most frequently distributed histone modification, and the main modification region is in the upstream and downstream region away from the TSSs (>500 bp); In TAZ, H3K4me3 is the most abundant histone modification, and it is distributed in a wide region near the TSSs (<1000 bp); In YAP, H3K4me3 is the most abundant histone modification, and mainly distributed in the downstream region away from the TSSs (>1000 bp). These results indicate that the distributions of histone modifications on the core genes of Hippo pathway have obvious type specificity and regional preference.

### Histone modification functional clusters of core genes in Hippo pathway

**Figure 3:**
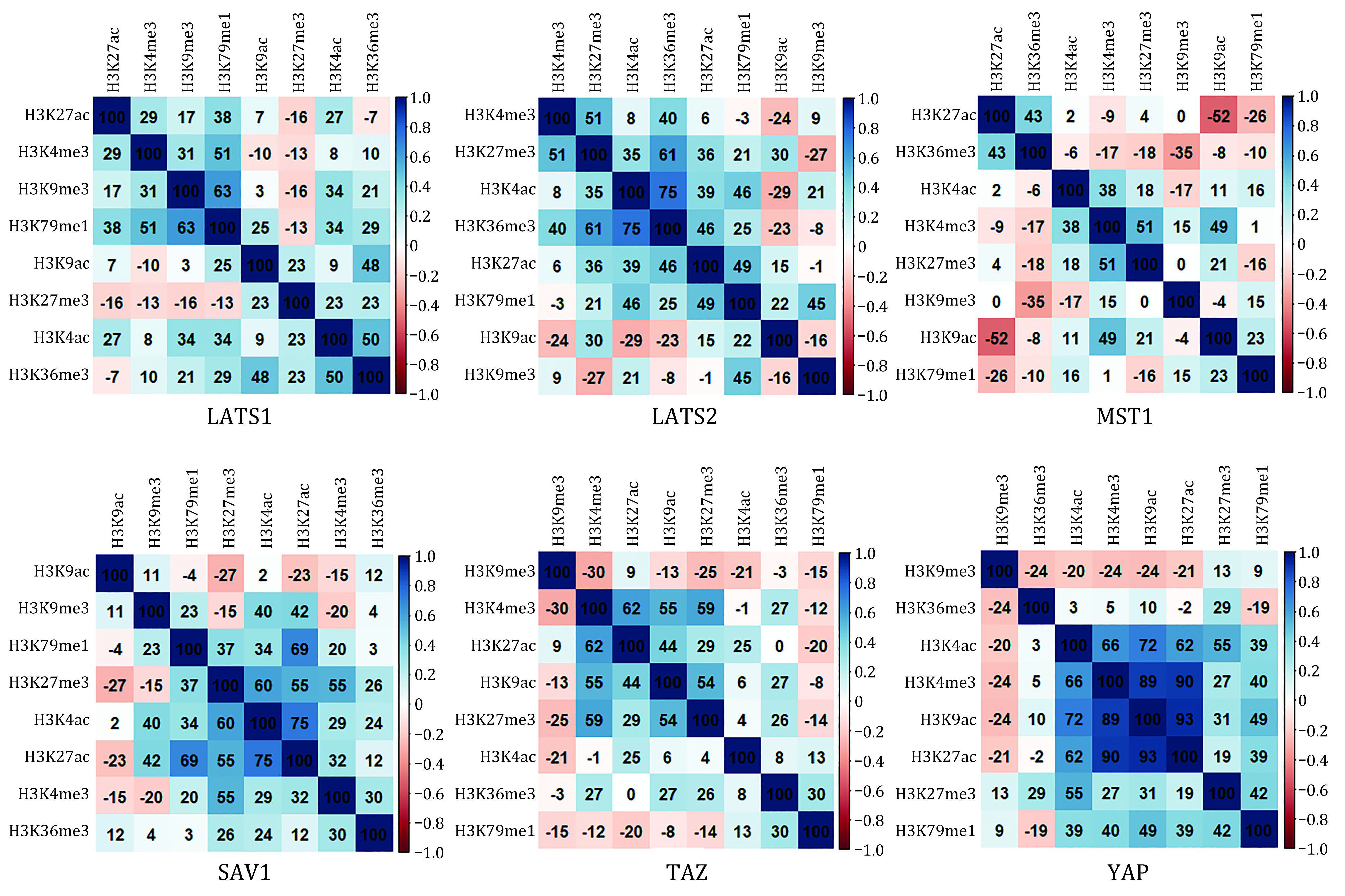
The heatmaps for Spearman rank correlations of histone modifications of the core genes in Hippo pathway

It has been proposed that histone modifications can cross-talk and that the robustness of a chromatin state can be reinforced by several modifications which have similar functions (Fischle *et al*. 2003; Li *et al*. 2020). In order to study the forms of histone modification functional clusters on the core genes of Hippo pathway, we calculated Spearman correlation coefficients between histone modifications in the flanking regions of TSSs of core genes in Hippo pathway, as shown in Fig. 3. It can be concluded from Fig. 3 that, in LATS1, the correlation coefficient between H3K79me1 and H3K9me3 is high (about 0.63); In LATS2, the correlation coefficient between H3K36me3 and H3K27me3 is high (about 0.61), at the same time, there is a high correlation coefficient between H3K36me3 and H3K4ac (about 0.75); There are no histone modification pairs with high correlations in MST1; In SAV1, there is a strong correlation between H3K27ac and H3K4ac (about 0.75), and the correlation coefficient between H3K27ac and H3K79me1 is high (about 0.69); In TAZ, the correlation coefficient between H3K4me3 and H3K27ac is high (about 0.62); In YAP, the correlation coefficients between H3K4ac and H3K4me3, and H3K9ac and H3K27ac are high (greater than 0.62), these four histone modifications tend to occur simultaneously and jointly regulate the expression of YAP. Thus, among the core genes of Hippo pathway, only gene YAP have been detected a histone modification functional cluster composed of H3K4ac, H3K4me3, H3K9ac and H3K27ac. Although there are histone modification pairs with high correlations in other genes, there are no obvious histone modification functional clusters.

### Distributions of histone modifications in the functional regions of core genes in Hippo pathway

In order to analyze the spatial distribution patterns of histone modifications in core genes of Hippo pathway in detail, we selected four functional regions, promoter, 5'UTR, genebody and 3'UTR to analyze the distribution patterns of histone modification in the four functional regions. Firstly, the total read count of each histone modification in the four functional regions of the core gene in Hippo pathway was obtained, as shown in Table 3. It can be seen from Table 3 that the distribution of the same histone modification in the functional regions of different core genes is quite different. Among them, H3K36me3 is the most widely distributed histone modification type in MST1, SAV1 and LATS2; H3K4ac is the most widely distributed histone modification type in LATS1 and LATS2; and H3K4me3 is the most widely distributed histone modification type in TAZ and YAP.

**Table 3:** Distributions of histone modifications in functional regions of core genes in Hippo pathway

	H3K4ac	H3K4me3	H3K9ac	H3K9me3	H3K27ac	H3K27me3	H3K36me3	H3K79me1
MST1	0	0	10	10	11	11	**70**	19
SAV1	30	4	26	21	24	4	**43**	23
LATS1	**358**	78	149	197	227	28	145	238
LATS2	38	6	8	28	14	25	**78**	19
TAZ	44	**90**	31	5	23	9	8	15
YAP	74	**105**	31	26	71	19	24	27
Firstly, the normalized read counts of each histone modification in each functional region were calculated, then the total read counts for all functional regions were calculated. The total read counts of the histone modifications which had the most distributions were indicated in bold.								

**Figure 4:**
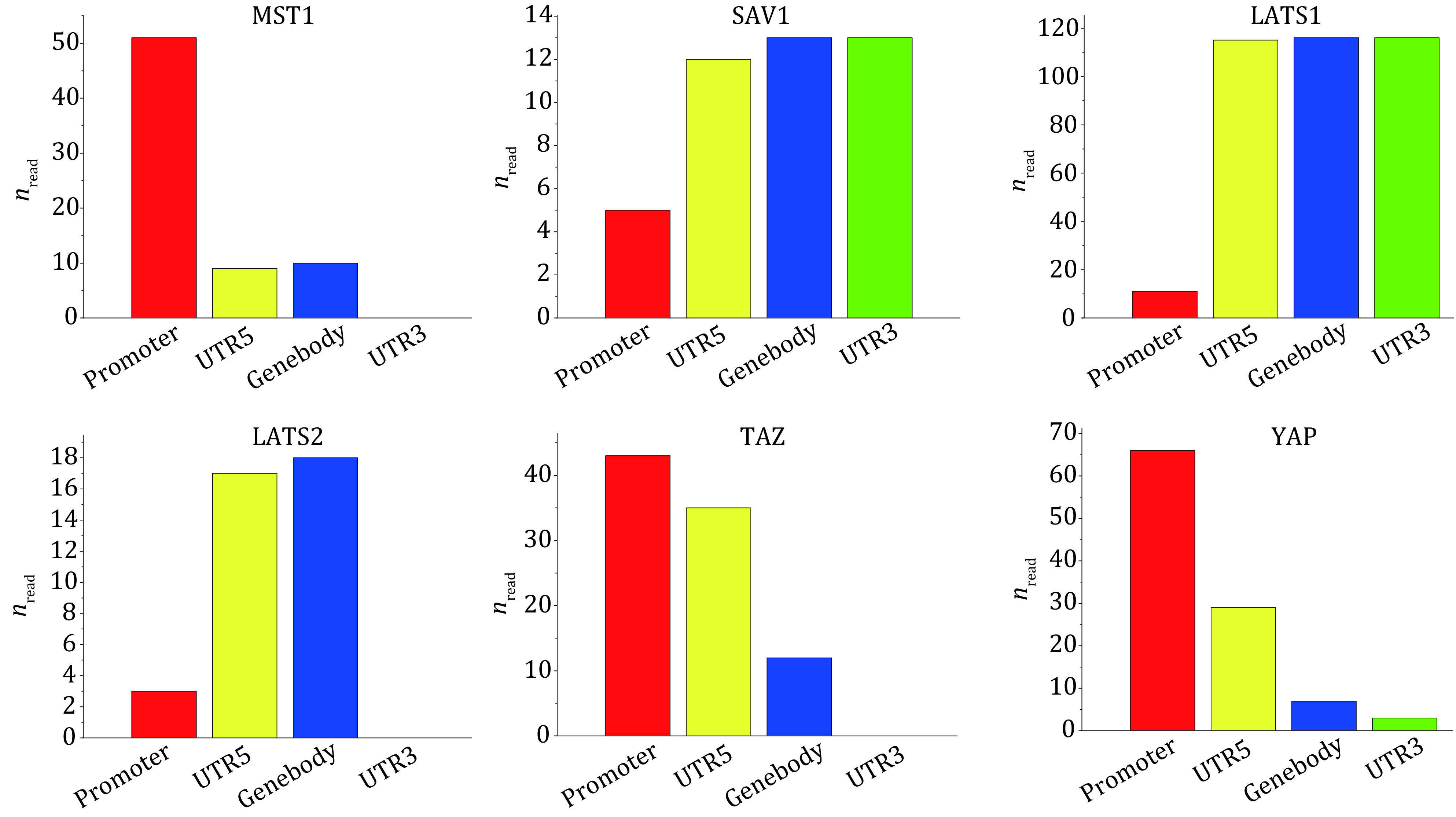
Histogram of histone modifications with the largest distributions in functional regions of core genes in Hippo pathway

In order to analyze the distributions of histone modifications in functional regions in detail, we obtained the distribution maps of histone modifications with the largest distributions on each core gene in promoter, 5'UTR, gene body and 3'UTR, as shown in Fig. 4. By analyzing the distribution maps, it can be concluded that the distributions of different histone modifications of different genes in the four functional regions are not exactly the same. In MST1, SAV1 and LATS2, H3K36me3 is the most widely distributed histone modification type. However, the distribution patterns of H3K36me3 in functional regions of three genes are different. In MST1 and LATS2, H3K36me3 is not found in 3'UTR; In MST1, the distribution of H3K36me3 in promoter is significantly higher than that in other regions; In LATS2, H3K36me3 is the most abundant in gene body, followed by 5'UTR; In SAV1, H3K36me3 is mostly distributed in gene body and 3'UTR, followed by 5'UTR. H3K4ac is the most widely distributed histone modification type of LATS1, and it is more distributed in 5'UTR, gene body and 3'UTR, and the least in promoter; In TAZ and YAP, H3K4me3 is the most frequently distributed histone modification, and H3K4me3 has the same distribution pattern in four functional regions, that is the decreasing distribution in promoter, 5'UTR, gene body and 3'UTR.

### Distributions of histone modifications in the functional regions of the whole genome

**Figure 5:**
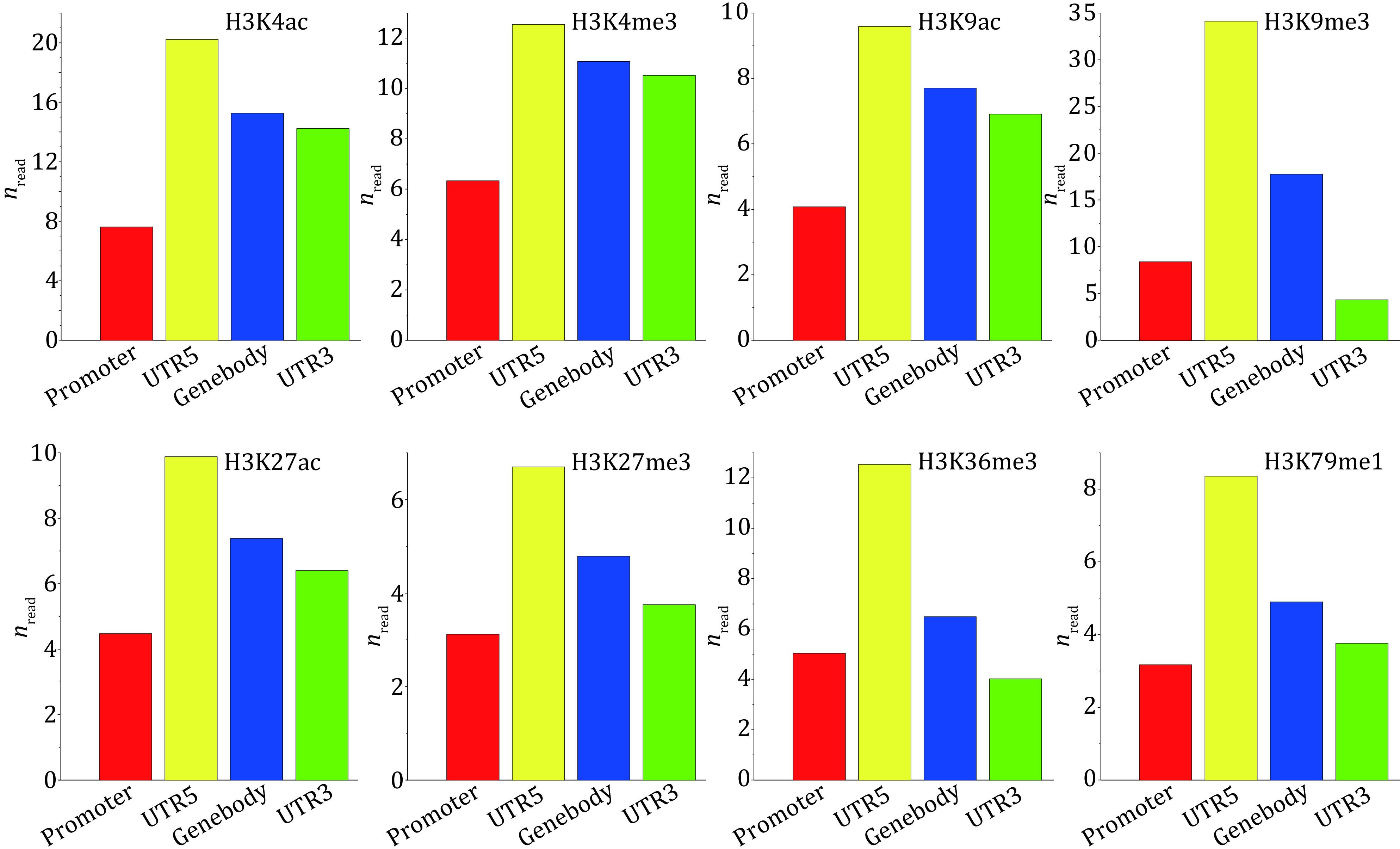
Histogram of histone modifications in functional regions of the whole genome

In order to further explore the spatial distribution patterns of histone modifications regulating the self-renewal process of embryonic stem cells, we got the distributions of histone modifications in four functional regions of the whole genome, as shown in Fig. 5. Through the analysis, it could be found that different from the distribution patterns of the specific genes, for the whole genome, the distributions of all histone modifications presents certain statistical regularity. All histone modifications mostly distribute in the 5'UTR, indicating that the downstream of transcription start site is an important region for histone modifications to regulate gene expression.

## CONCLUSION

By studying the spatial distribution pattern of histone modification in Hippo pathway, we found that the distributions of eight histone modifications had obvious type specificity and location preference on the 19 Hippo pathway genes, in which H3K4me3 and H3K36me3 played the most important regulatory role. At the same time, the distribution patterns of histone modifications in the flanking TSS region, promoter, 5'UTR, gene body and 3'UTR of YAP, TAZ, LATA1/2, MST1 and SAV1 were obtained, which indicated that the distributions of histone modifications had obvious gene specificity and spatial preference. Different from the distribution patterns of the core genes, the distributions of histone modifications have obvious statistical regularity for the whole genome. Histone modifications are mostly distributed in 5'UTR, which is the main target region of histone modifications for the whole genome. Through the correlation analysis between histone modifications, we detected one histone modification functional cluster in YAP, composed of H3K4ac, H3K4me3, H3K9ac and H3K27ac.

In this paper, the spatial distribution patterns of histone modifications on Hippo pathway genes were obtained, which provided a new theoretical reference for elucidating the mode of histone modifications regulating the gene expression of Hippo pathway, and for revealing the molecular regulatory mechanism of histone modifications affecting the self-renewal of embryonic stem cells by regulating the Hippo pathway.

## MATERIALS AND METHODS

### Data sources

The ChIP-seq data of eight kinds of histone modifications (H3K4ac, H3K4me3, H3K9ac, H3K9me3, H3K27ac, H3K27me3, H3K36me3 and H3K79me1) for the human H1 cell line were downloaded from the NIH Roadmap Epigenomics project (http://www.ncbi.nlm.nih.gov/geo/roadmap/epigenomics/).

The data of RefSeq genes of human genome were downloaded from UCSC (http://genome.ucsc.edu/), which contain the gene names, chromosomes, strands, transcription starts, transcription ends, translation starts, translation ends, exon counts, exon starts, and exon ends. The existing studies showed that the distribution preference of the same histone modification in different regions of the genome was different (Takahashi and Yamanaka 2006). Therefore, according to the RefSeq genes of human genome, the genes were divided into four different functional regions: promoter, 5'UTR, gene body and 3'UTR, and the flanking regions of transcription start sites were selected to explore the spatial distribution patterns of histone modifications.

According to the DAVID database (https://david.ncifcrf.gov/), 19 Hippo pathway genes were extracted, composed of DCHS1, DCHS2, FAT1, FAT2, FAT3, FAT4, FRMD6, WWC1, NF2, MST1, SAV1, LATS1, LATS2, TAZ, TEAD1, TEAD2, TEAD3, TEAD4 and YAP.

### Calculation of histone modification signal profiles

#### The read counts of histone modifications in the flanking regions of transcription start sites

In order to get the signal profiles of eight histone modifications in the flanking regions of transcription start sites (TSS ± 2000 bp), the normalized read counts were calculated based on the ChIP-seq data. In order to eliminate the influence of the length of regions and the sequencing depth, we defined the computational formulas of the normalized read counts as Eq. 1 and 2 according to computing method of RPKM. The DNA regions (TSS ± 2000 bp) were divided into 20 bins of 200 bp in size, and for each bin the read counts of histone modifications were normalized by bin length and the total read counts were calculated in the measurement using the following Eq. 1.

1 $ {n}_{\mathrm{r}\mathrm{e}\mathrm{a}\mathrm{d}}={10}^{9}\times {n}_{\mathrm{b}\mathrm{i}\mathrm{n}}/({l}_{\mathrm{b}\mathrm{i}\mathrm{n}}\times {N}_{\mathrm{r}\mathrm{e}\mathrm{a}\mathrm{d}})\;, $

where $ {n}_{\mathrm{r}\mathrm{e}\mathrm{a}\mathrm{d}} $ represented the normalized read counts, $ {n}_{\mathrm{b}\mathrm{i}\mathrm{n}} $ was the read counts that located in this bin, $ {l}_{\mathrm{b}\mathrm{i}\mathrm{n}} $ was the length of the bin, and $ {N}_{\mathrm{r}\mathrm{e}\mathrm{a}\mathrm{d}} $ was the total read counts in the measurement.

#### The read counts of histone modifications in the functional regions

Each gene was divided into promoter, 5'UTR, gene body and 3'UTR regions based on the annotation of the RefSeq genes. The read counts of histone modifications were normalized by the length of the functional regions and the total read counts in the measurement, showing as Eq. 2, in which $ {n}_{\mathrm{r}\mathrm{e}\mathrm{a}\mathrm{d}} $ represented the normalized read counts, $ {n}_{\mathrm{r}\mathrm{e}\mathrm{g}\mathrm{i}\mathrm{o}\mathrm{n}} $ was the total read counts that located in this functional region, $ {l}_{\mathrm{r}\mathrm{e}\mathrm{g}\mathrm{i}\mathrm{o}\mathrm{n}} $ was the length of this functional region, and $ {N}_{\mathrm{r}\mathrm{e}\mathrm{a}\mathrm{d}} $ was the total read counts in the measurement.

2 $ {n}_{\mathrm{r}\mathrm{e}\mathrm{a}\mathrm{d}}={10}^{9}\times {n}_{\mathrm{r}\mathrm{e}\mathrm{g}\mathrm{i}\mathrm{o}\mathrm{n}}/({l}_{\mathrm{r}\mathrm{e}\mathrm{g}\mathrm{i}\mathrm{o}\mathrm{n}}\times {N}_{\mathrm{r}\mathrm{e}\mathrm{a}\mathrm{d}})\;. $

#### The correlations between different histone modifications

It has been reported that histone modifications can occur in combination to form a “histone code” to affect gene transcription (Ucar *et al*. 2011). The Spearman rank correlations between different histone modifications were calculated to detect the functional histone modification clusters in the core genes of Hippo pathway. For each histone modification, the normalized read counts in each bin were obtained. Then a 20- dimensional vector was built for each histone modification, vector element *m_i_* (*I* = 1, …, 20) represented the normalized read counts in bin *i*. The Spearman rank correlations for each pair of histone modifications were calculated based on the above vector, a $ 8\times 8 $ matrix was obtained, in which the matrix element *t_ij_* (*I* = 1, …, 8, *j* = 1, …, 8) was the Spearman rank correlation between histone modification *i* and histone modification *j*. Based on this matrix, a heatmap was plotted.

## Conflict of interest

Wenxia Su, Dimeng Zhang, Qiang Zhang and Qianzhong Li declare that they have no conflict of interest.
